# Periprosthetic Joint Infection Following Reverse Shoulder Arthroplasty Treated With Continuous Local Antibiotic Perfusion: A Case Report

**DOI:** 10.7759/cureus.49193

**Published:** 2023-11-21

**Authors:** Ryosuke Mashiko, Taku Hatta, Chiharu Nagashima

**Affiliations:** 1 Orthopedic Surgery, Joint Surgery, Sports Clinic Ishinomaki, Ishinomaki, JPN; 2 Traumatology and Reconstructive Surgery Center, Aizu Chuo Hospital, Aizuwakamatsu, JPN

**Keywords:** intra-soft tissue antibiotic perfusion, surgery, reverse shoulder arthroplasty, prosthetic joint infection, continuous local antibiotic perfusion

## Abstract

Prosthetic joint infection (PJI) is a crucial complication of reverse shoulder arthroplasty (RSA). Continuous local antibiotic perfusion (CLAP) with a high-concentration antimicrobial pharmacy administration method has recently received attention owing to its effectiveness in the treatment of bone and soft tissue infections. We herein report a case of PJI following RSA that was successfully treated with CLAP without removal of the entire implant. A 73-year-old woman with comorbidities of diabetes mellitus and hypertension underwent RSA. The wound was found to be swollen eight weeks after RSA, and purulent content that was positive for *Propionibacterium granulosum* was identified. Blood samples indicated a mildly elevated inflammatory response. With a diagnosis of PJI spread from the intra-articular to subcutaneous regions without implant loosening, the patient underwent surgical treatment nine weeks after RSA. The contaminated tissues were thoroughly debrided, and the prosthetic joint was preserved by replacing the glenosphere and polyethylene liner. Intra-soft tissue antibiotic perfusion (iSAP) tubes and effluent drains were placed intra-articularly and subcutaneously, and gentamicin was infused continuously for 12 days. In addition, ceftriaxone and rifampicin were administered. The patient was subsequently treated with minocycline and sulfamethoxazole/trimethoprim or clindamycin for eight weeks. The inflammatory reaction became negative six weeks postoperatively, and the patient had no recurrence at 15 months postoperatively. Treatment of PJI is considered a long-lasting, challenging process.

This case report supports the feasibility of using CLAP in the treatment of PJI.

## Introduction

Reverse shoulder arthroplasty (RSA) has been recognized as a reliable surgical procedure that provides satisfactory clinical outcomes, with expanded surgical indications for various shoulder disorders, including cuff tear arthropathy, irreparable rotator cuff tear, bone neoplasm, rheumatoid arthritis, osteoarthritis, and comminuted proximal fracture in elderly patients [1]. Periprosthetic joint infection (PJI) remains a crucial complication following RSA, potentially causing pain, decreased functional recovery, prolonged hospitalization, requirement for additional surgery, and/or increased mortality [2]. PJI has been reported in 0.9%-1.7% of cases following RSA, and complicated and multiple surgeries may be needed for infection control and subsequent functional restoration [3,4].

Continuous local antibiotic perfusion (CLAP), which continuously circulates antibiotic agents throughout the lesion, has recently received attention as an alternative treatment for severe fracture-related infections with retaining implants [5,6]. Intra-medullary antibiotic perfusion (iMAP) for intra-medullary infection or intra-soft tissue antibiotic perfusion (iSAP) for soft-tissue infection has the advantage of providing a high local concentration of antibacterial agents in infected regions. Several case reports have demonstrated satisfactory outcomes in patients with fracture-related infection following open fracture and/or surgical intervention [7,8]. In contrast, there have been few clinical reports on the use of CLAP for PJI. To our knowledge, no reports have described CLAP for cases of PJI in the shoulder joint.

We herein report a case of PJI following RSA that was successfully treated via CLAP while preserving the prosthetic joint.

## Case presentation

**Figure 1:**
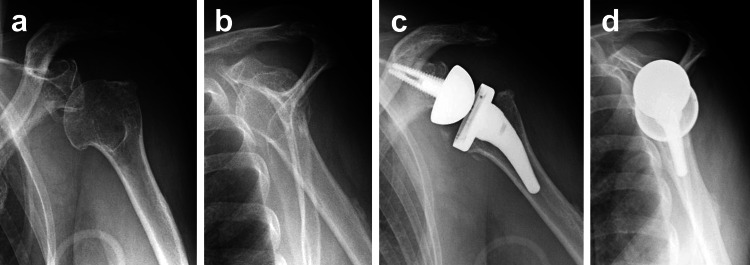
Plain radiographs The patient underwent reverse shoulder arthroplasty (RSA) for residual fracture-dislocation of the shoulder. Preoperative (a: anteroposterior image, b: lateral image) and postoperative (c: anteroposterior image, d: lateral image) radiographs.

A 73-year-old woman visited our institution after falling and impacting her left shoulder. The patient had comorbidities of diabetes mellitus and hypertension. Radiologically, the patient was diagnosed with shoulder dislocation with anterior rim fracture of the glenoid. Since an arthroscopic procedure using soft anchors resulted in failed stability with persistent pain and restricted motion, the patient was scheduled to undergo RSA three weeks after the initial trauma (Figures 1a, 1b). RSA was performed using the AEQUALIS Ascend system (Stryker Ltd., Kalamazoo, MI, USA) via the deltopectoral approach (Figures 1c, 1d).

**Figure 2:**
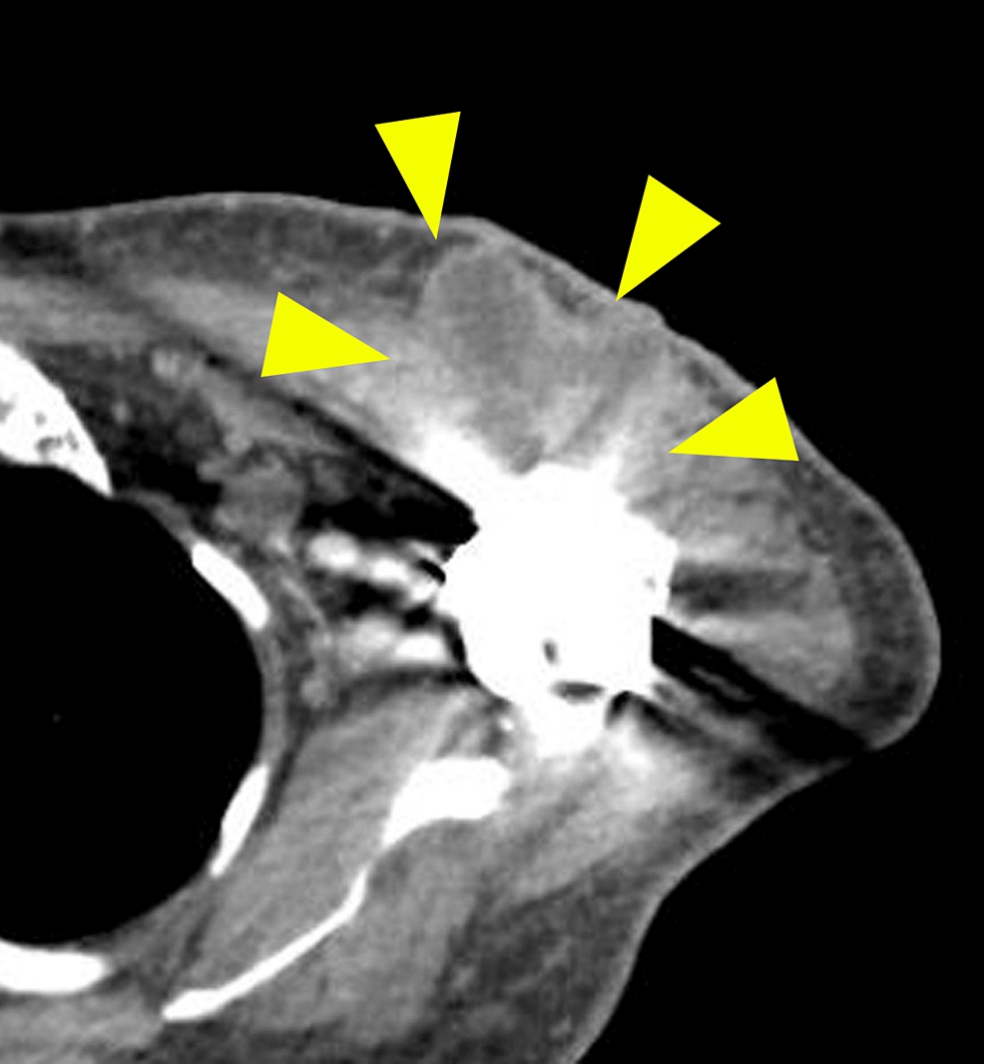
Enhanced computed tomography Yellow arrowheads indicate an infected lesion with rim enhancement spreading subcutaneously to the periprosthetic space.

Postoperatively, shoulder pain with a swollen wound was detected eight weeks after RSA. Computed tomography (CT) revealed a cystic lesion spreading from the shoulder joint to the subcutaneous tissues (Figure 2). The aspirated purulent fluid contained Gram-positive bacteria, and culture examination for three days identified *Propionibacterium granulosum*. Before identifying the bacteria, no antibacterial agents were administered.

**Figure 3:**
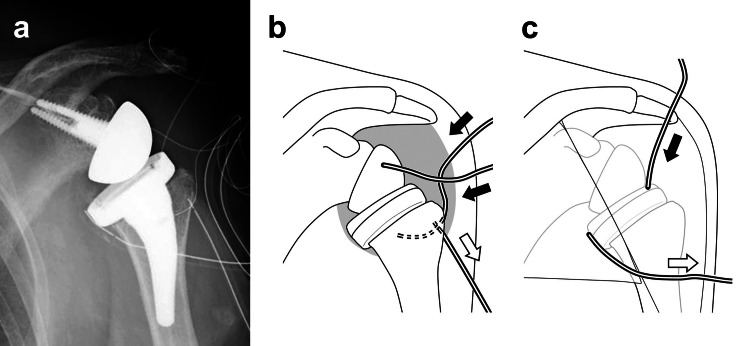
Postoperative radiograph and schematic illustrations Postoperative anteroposterior image (a) demonstrates the placement of intra-soft tissue antibiotic perfusion (iSAP) tubes and effluent drains for continuous local antibiotics perfusion (CLAP). Schematic illustrations represent iSAP tubes (black arrows) and effluent drains (white arrows); two iSAP tubes were placed in the periprosthetic space (b), and one iSAP tube was placed subcutaneously (c).

The patient was diagnosed with PJI and underwent surgical treatment nine weeks after RSA. Intraoperatively, contaminated soft tissues were thoroughly debrided using high-power water via the VERSAJET Hydrosurgery System (Smith & Nephew Ltd., London, UK). The prosthetic joint was preserved simply by replacing the glenosphere and polyethylene liner. For the CLAP treatment, two iSAP tubes and an effluent drain were placed in the periprosthetic space. In addition, an iSAP tube and an effluent drain were placed subcutaneously as well (Figures 3a-3c).

**Figure 4:**
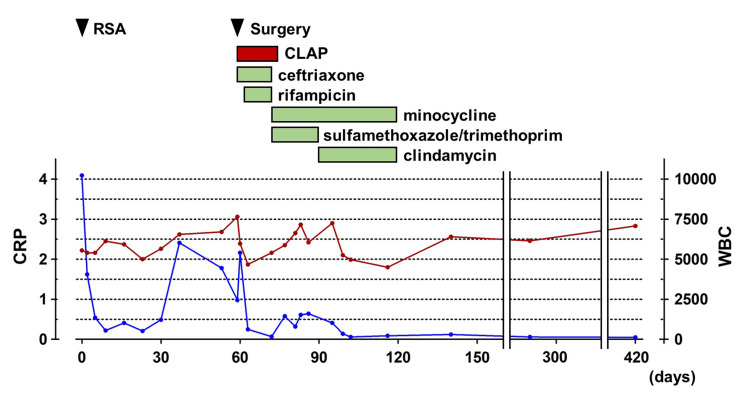
Timeline to represent continuous local antibiotics perfusion (CLAP) and systemic administration of antibiotics The graph represents the trend in levels of C-reactive protein (CRP, blue line) and the number of white blood cells (WBCs, red line).

Gentamicin was continuously infused from each iSAP tube at 2.4 mg/h for 12 days. According to the antimicrobial susceptibility testing examined before the surgery, ceftriaxone and rifampicin were administered intravenously and orally respectively for one week, and subsequently, minocycline and sulfamethoxazole/trimethoprim or clindamycin were administered orally for eight weeks (Figure 4). The inflammatory reaction became negative six weeks postoperatively, and the patient had no recurrence at 15 months postoperatively.

## Discussion

We encountered a case of PJI following RSA treated with CLAP. To our knowledge, there have been no clinical reports demonstrating the application of CLAP for PJI of the shoulder joint. The current case of PJI in a shoulder that had undergone RSA was successfully treated with CLAP. Notably, treatment for infection control can be achieved by retaining the prosthetic joint. The current case report indicates that CLAP can be useful for PJI, with the advantages of minimal functional deficiency and medical cost.

Regarding the importance of PJI in patients following RSA, several studies have demonstrated risk factors for PJI, such as sex, smoking history, iron-deficiency anemia, pathological weight loss, and obesity [4,9]. Regarding the treatment of PJI, various surgical procedures, including irrigation and debridement with/without implant retention, implantation of a cement spacer or resection arthroplasty, and one- or two-stage revision [10]. Although the optimal treatment strategies for PJI remain controversial, the relative advantages of one-stage versus two-stage revision have been investigated. In the literature, one-stage revision has been reported to have several advantages, including reduced reinfection rates, reduced medical costs, shorter hospital stays, better functional outcomes, and overall shorter antibiotic protocol administration, leading to less damage to soft tissues and lower rates of surgical comorbidity [11]. However, two-stage revision has shown benefits of its own, including removal of all hardware in which a biofilm may form, soft tissue rest, less surgical time in each intervention, and allowance of careful planning for secondary joint reconstruction [12]. Although the comparative data were insufficient, a recent systematic review including 711 shoulders with PJI demonstrated a lower reinfection rate for one-stage revision (1.14%) than for two-stage revision (8.81%) and a lower complication rate for one-stage revision (6.11%) than for two-stage revision (21.26%) [13].

 CLAP has been recently focused on the treatment of persistent soft-tissue infections, especially fracture-related infections [5,6]. Regarding the administration of antibiotics, it has been recognized that it is difficult to maintain high concentrations to exceed the minimal biofilm eradication concentration (MBEC), rather than to focus on the minimum inhibitory concentration (MIC). The MBEC should be defined as a concentration that is 100-1000 times the MIC [14]. Therefore, surgeons may have to determine the removal of all implants if insufficient concentrations of MBEC with intravenously administered antibiotic agents are considered the sequence of residual, uncontrollable PJI.

CLAP has the advantage of allowing the administration of any concentration of antibiotic. Previous clinical reports have successfully controlled fracture-related infections while retaining implants [7,8]. Although there is insufficient evidence to support CLAP for the treatment of PJI, potential benefits, including effective antibiotic administration at the infected site with retaining prosthesis, decreased medical costs, and a shortened hospital stay, can be mentioned. However, surgical indications should be carefully determined, especially in cases with chronic PJI in which advanced infiltration throughout the implants may be observed. In addition, the generalizability of applying CLAP procedures such as the placement of iSAP tubes should be noted.

## Conclusions

The current case report demonstrated the successful management of PJI using CLAP. We described CLAP as a novel treatment option for PJI with the advantage of maintaining a sufficient concentration of the administered antibiotic agent in the infected region. Notably, the current treatment for infection control can be achieved by retaining the prosthetic joint. We indicate that the application of CLAP with surgical debridement and implant retention may be a feasible option to maintain shoulder function in patients with PJI.
